# Baseline Triglyceride Level Affected the Efficacy of Vildagliptin in Treating Type 2 Diabetes: A Post Hoc Analysis of the VISION Study

**DOI:** 10.1155/2019/9347132

**Published:** 2019-08-08

**Authors:** Lingli Zhou, Xiaoling Cai, Yingying Luo, Fang Zhang, Linong Ji

**Affiliations:** Peking University People's Hospital, 11 Xizhimen Street, Xicheng District, Beijing 100044, China

## Abstract

Identifying factors that may impact vildagliptin's efficacy could contribute to individualized treatment for patients with type 2 diabetes. In the current study, we aimed to assess the correlation between patient baseline triglyceride (TG) and efficacy of vildagliptin in Chinese patients with type 2 diabetes in a post hoc analysis of the VISION study. TG-based subgroup analysis was performed to evaluate baseline TG's impact on the decrease of glycated hemoglobin (HbA_1c_) in patients receiving vildagliptin plus low-dose metformin (VLDM) vs. high-dose metformin (HDM). Additionally, multivariate linear regression was performed to assess the association between baseline TG and HbA_1c_ reduction at weeks 12 and 24 for patients receiving VLDM vs. HDM. For patients receiving VLDM, baseline TG ≤ 2.03 mmol/L was associated with significantly greater HbA_1c_ reduction vs. TG > 2.03 mmol/L at week 12, but not at week 24. Additionally, multivariate linear regression analysis revealed a significant independent association and an association short of statistical significance between patient baseline TG and the HbA_1c_-reducing efficacy of VLDM at weeks 12 (*P* < 0.001) and 24 (*P* = 0.082), respectively, while such association was absent for HDM. Collectively, baseline TG was an independent predictive factor for the efficacy of a dipeptidyl peptidase-IV in treating type 2 diabetes during its initial use.

## 1. Introduction

Vildagliptin is a dipeptidyl peptidase-IV (DPP-4) inhibitor approved for the treatment of type 2 diabetes mellitus (T2DM). By inhibiting DPP-4, vildagliptin prevents the degradation of glucagon-like peptide-1 (GLP-1) and glucose-dependent insulinotropic polypeptide (GIP) and increases the level of biological active, intact plasma GLP-1, and GIP-1; as a result, it could restore or improve pancreatic *α*- and *β*-cell sensitivity to glucose and suppress glucagon release [1]. Clinical studies found that vildagliptin in combination with metformin resulted in better glycemic control than high-dose metformin alone [2–5]. Despite a better glucose control achieved by vildagliptin plus low-dose metformin (VLDM) than high-dose metformin (HDM) in our pervious VISION study, it showed 47.1% of patients have not yet satisfied the recommended target glycated hemoglobin (HbA_1c_) level of ≤6.5% after a six-month treatment by VLDM [5]. Identifying factors that impact the efficacy of vildagliptin could contribute to individualized treatment for T2DM.

It has been reported that nonesterified fatty acid (NEFA) treatment decreased GLP-1 receptor (GLP-1R) expression in rodent insulinoma cell lines and isolated islet and led to impaired GLP-1 signaling. Further, lowering of plasma TGs by fibrates increased the efficacy of the DPP-4 inhibitor des-fluoro-sitagliptin in *db/db* mice [6]. Interestingly, Duca et al. found that high-energy (HE)/high-fat (HF) feeding led to significant downregulation of GLP-1R expression in the vagal nodose ganglia of obesity-prone (OP) but not obesity-resistant (OR) rats and that the combination of HE/HF feeding and the OP phenotype led to reduced endogenous GLP-1 and GLP-1R activation [7]. These findings of animal studies suggested the possibility that patient baseline TG levels could impact the efficacy of incretin-based therapies. A recent study found that markers of higher insulin resistance, including triglyceride, are consistently associated with reduced glycemic response to DPP-4 inhibitors in T2DM patients with DPP-4 inhibitor monotherapy [8]. However, it is still unknown whether the inverse relationship between triglyceride and glucose control is solely contributed to DPP-4 inhibitor treatment or due to drugs targeting on insulin resistance. Therefore, the current post hoc analysis of the VISION study was undertaken to determine whether a patient baseline TG level was associated with the HbA_1c_-reducing effect of VLDM and HDM.

## 2. Methods

### 2.1. Design

Details of the design and method of the 24-week, phase 4, multicenter, randomized, open-label, prospective, parallel-group VISION study have been described previously [5]. Briefly, patients with T2DM inadequately controlled with metformin 1000 mg daily were divided 1 : 1 : 1 : 1 into four prespecified subgroups based on age and body mass index (BMI). Patients in each subgroup were randomized 5 : 1 to receive either vildagliptin (50 mg twice daily) plus metformin [500 mg twice daily; vildagliptin and low-dose metformin (VLDM) group] or metformin uptitration [1000 mg twice daily; high-dose metformin (HDM) group]. The primary endpoint was change in glycated hemoglobin (HbA_1c_) from baseline at week 24. This clinical trial was designed, conducted, and reported in accordance with Good Clinical Practice (GCP) and the Declaration of Helsinki. The study protocol has also been approved by the Independent Ethics Committee (IEC) or Institutional Review Board (IRB) of each participating study center.

### 2.2. Post Hoc Analysis Procedures

The analysis of the VISION data was conducted on the full analysis set (FAS) population (patients who received at least one dose of study drug and had at least one primary or secondary efficacy evaluation after baseline) with last observation carried forward (LOCF). The VLDM and HDM groups were further grouped into 3 tertiles/subgroups based on their baseline TG levels: “TG < 1.28 mmol/L” (1^st^ tertile), “1.28 mmol/L ≤ TG ≤ 2.03 mmol/L” (2^nd^ tertile), and “TG > 2.03 mmol/L” (3^rd^ tertile). HbA_1c_ changes from baseline (*Δ*HbA_1c_) at weeks 12 and 24 served as endpoints that were assessed in all subgroups. Correlations of baseline TG and HbA1c reduction at weeks 12 and 24 were also analyzed.

### 2.3. Statistical Analysis

The mean ± standard deviation (SD) was used to describe *Δ*HbA_1c_ at weeks 12 and 24. Since total cholesterol and TG did not follow a normal distribution, the values were log transferred (e.g., log (baseline total cholesterol) and log (baseline TG)). Baseline characteristics were compared among the three subgroups by the one-way analysis of variance (ANOVA). Both univariate and multivariate analyses were performed for the intersubgroup comparison of *Δ*HbA_1c_. The univariate analysis used the TG subgroups as the variable and baseline HbA_1c_ as the covariate. The multivariate analysis used the TG subgroups as the variable; covariates included baseline HbA_1c_, BMI, systolic blood pressure (SBP), total cholesterol (TC), ALT, creatinine, and duration of T2DM, whether the patients took lipid-lowering medication and whether the patients took *αβ*-blockers or *β*-blockers. Patients' compliance with the treatment was also used as one of the covariates.

Correlations between the patient baseline TG level and *Δ*HbA_1c_ at weeks 12 and 24 for the VLDM and HDM groups were further assessed using multivariate linear regression analysis (an analysis of covariance (ANCOVA)), wherein *Δ*HbA_1c_ at weeks 12 and 24 was a dependent variable. Independent variables included patient baseline demographics and clinical characteristics such as age, gender, baseline alanine transaminase (ALT), 120 min postprandial blood glucose (PPG), fast plasma glucose (FPG), serum creatinine, HbA_1c_, BMI, duration of type 2 diabetes, log (baseline total cholesterol), log (baseline TG), systolic blood pressure (SBP), and diastolic blood pressure (DBP), whether the patients took lipid-lowering medication and whether the patients took *αβ*-blockers or *β*-blockers. Patients' compliance with the treatment was also included as one of the independent variables.

The statistical significance was accepted with a *P* value < 0.05. Statistical analyses were performed using SAS version 9.2.

## 3. Results

### 3.1. Characteristics of Participants

Data from the FAS population that included 2,501 patients in the VLDM arm and 484 patients in the HDM arm were analyzed. Patients in the VLDM and HDM arms were grouped into 3 subgroups according to their baseline TG: “TG < 1.28 mmol/L” (1^st^ tertile; 826 and 172 patients in VLDM and HDM, respectively), “1.28 mmol/L ≤ TG ≤ 2.03 mmol/L” (2nd tertile; 855 and 156 patients in VLDM and HDM, respectively), and “TG > 2.03 mmol/L” (3rd tertile; 819 and 155 patients in VLDM and HDM, respectively), and their baseline demographics and clinical characteristics are summarized in Table 1. For patients in both the VLDM and the HDM arms, there were baseline differences among the 3 TG-based subgroups (Table 1).

### 3.2. Impact of Baseline TG on HbA_1c_ Change from Baseline and Predictive Value of Baseline TG

For the VLDM group, at week 12, patients in the 1^st^, 2^nd^, and 3^rd^ tertiles had a mean *Δ*HbA_1c_ of -0.55% ± 0.816%, -0.52% ± 0.754%, and -0.48% ± 0.789%, respectively, and at week 24, they had a mean *Δ*HbA_1c_ of -0.57% ± 0.905%, -0.55% ± 0.858%, and -0.51% ± 0.882%, respectively. Univariate analysis revealed significant differences in HbA_1c_ reduction across the 3 subgroups at weeks 12 and 24 (*P* = 0.007 and *P* = 0.025, respectively) (Table 2). Particularly, univariate analysis showed that patients in both the 1^st^ and 2^nd^ tertiles had significantly greater HbA_1c_ reduction than patients in the 3^rd^ tertile at weeks 12 and 24 (*P* < 0.05 for both) (Table 2). Multivariate analysis reveals the same significant differences in HbA_1c_ reduction across the 3 subgroups at week 12 (*P* = 0.008) and that patients in both the 1^st^ and 2^nd^ tertiles had significantly greater HbA_1c_ reduction than patients in the 3^rd^ tertile at week 12 (*P* < 0.05); however, at week 24, multivariate analysis failed to reveal any significant difference in HbA_1c_ reduction among the 3 TG-based groups (*P* = 0.113) (Table 2).

For patients receiving HDM, no significant difference in HbA_1c_ reduction was found among the 3 TG-based subgroups at both week 12 and week 24 (*P* > 0.05) (Table 2).

Multivariate linear regression analysis further revealed significant association and an association short of statistical significance between Log (baseline TG) and *Δ*HbA_1c_ at week 12 (regression coefficient: 0.1083 [95% CI: 0.0537, 0.1629]; *P* < 0.001) and week 24 (regression coefficient: 0.0551 [95% CI: -0.0071, 0.1173]; *P* = 0.082), respectively, for patients in the VLDM group (Table 3), but not for patients in the HDM group.

## 4. Discussion

This post hoc analysis of the VISION study revealed that patient baseline TG was an independent predictive factor for the HbA_1c_-lowering efficacy of VLDM at week 12, although such predictive effect diminished as the VLDM treatment continued and became insignificant at week 24. Since such association was lacking for HDM, it could be deduced that it was vildagliptin's efficacy that was affected by patients' baseline TG. Our study reported a statistically significant association between the patient baseline TG level and a DPP-4 inhibitor's efficacy in glycemic control during its early use. This finding is just agreed with previous animal and human studies [2–4, 8]. Our finding was further supported by the recently published study (Tanabe et al.) reporting that hypertriglyceridemia was an independent predictor for the efficacy of the GLP-1 analog liraglutide [9]. Our analysis further expanded their findings by TG-based subgroup analysis to examine the association between patient baseline TG and treatment effects of vildagliptin.

Importantly, we found this relationship did not exist in patients treated with metformin alone. The underlying mechanisms that the differences responded between DPP-4 inhibitor and metformin are still unknown. The differences of the pharmacological function between two drugs may partially explain the divergence. Unlike vildagliptin that prevents GLP-1 and GIP-1 degradation and increases the level of biological active, the efficacy of metformin depends on its capacity to reduce hepatic glucose production and improve metabolic parameters [10]. Duca et al. suggested that high-energy/high-fat feeding combined with an obesity-prone phenotype led to reduced endogenous GLP-1 and GLPR activation [7], suggesting that a high circulation TG level may decrease endogenous GLP-1R activation, therefore affecting the treatment efficacy of the DPP-4 inhibitor. That lowering of the TG level by fibrates which improved the efficacy of the DDP-4 inhibitor in a *db/db* mouse model verified this hypothesis [6]. A further intervention clinical study should be performed.

We speculated that for those patients with TG > 2.03 mmol/L, adding a lipid-lowing medication to the vildagliptin or other DDP-4 inhibitor-containing antidiabetic regimen may improve treatment efficacy at least during their early treatment period. The current American Diabetes Association guideline on standards in medical care in diabetes recommends lifestyle change and dietary for diabetic patients with hypertriglyceridemia, evaluation for the second cause, and considering medical therapy for patients with TG > 5.7 mmol/L and immediate pharmacological therapy only for patients with severe hypertriglyceridemia (TG > 11.4 mmol/L) to reduce the risk of pancreatitis [11]. However, our results suggested that for a patient taking vildagliptin or possibly other DPP-4 inhibitors with TG > 2.03 mmol/L, lipid-lowing pharmacological intervention during the early use of these DPP-4 inhibitors might be beneficial. More studies are needed to explore this position and also to validate the clinical relevance of our results in view of the fact that the difference in *Δ*HbA_1c_ between the 1st and the 3rd TG-based tertiles was small although the fact that both our multivariate analysis based on the 3 TG subgroups and our multivariate linear regression analysis produced consistent results suggested the robustness of our findings.

Our analysis had certain limitations. Our sample size for HDM was relatively small, and there is the possibility of insufficient statistic power to detect any significant association between baseline TG and HDM's efficacy especially in view of our results that both VLDM and HDM led to the greatest HbA_1c_ reduction in the 1st TG-based tertile and the smallest reduction in the 3 tertile (Table 2). Another limitation was the relatively short treatment duration of the VISION study; therefore, we cannot ascertain the impact of baseline TG on the HbA_1c_-reducing effect of vildagliptin beyond the 24-week treatment period. A prespecified, properly randomized, large-scale, longer-term study is currently being planned. Additionally, the present study demonstrated that the impact of baseline TG on treatment effects of vildagliptin decreased as the vildagliptin use continued. Possible reason(s) for such change over time is currently unclear. Nevertheless, our preliminary finding provided a new evidence of a precision approach for the DPP-4 inhibitor in treating T2DM.

In summary, our results suggested that the patient baseline TG level affected and was an independent predictive factor for the efficacy in glycemic control of vildagliptin, not metformin, for patients with T2DM.

In conclusion, our analysis is the first to report that the patient baseline TG level was an independent predictive factor for the glycemic control effect of a DPP-4 inhibitor, but not metformin, in patients with T2DM during its initial use.

## Figures and Tables

**Table 1:** Patients' baseline demographics and clinical characteristics (FAS) according to the 3 TG-based tertiles.

	Vildagliptin+metformin (VLDM)				Metformin (HDM)			
	1^st^ tertile *n* = 826	2^nd^ tertile *n* = 855	3^rd^ tertile *n* = 919	*P* value	1^st^ tertile *n* = 172	2^nd^ tertile *n* = 156	3^rd^ tertile *n* = 155	*P* value
Age (years)	58 (11)	57 (11)	55 (11)	<0.0001	58 (10)	56 (12)	55 (11)	0.0555
Male, *n* (%)	462 (55.9)	419 (49.0)	479 (58.5)	<0.0001	79 (45.9)	73 (46.8)	87 (56.1)	0.1314
Weight (kg)	67.2 (11.1)	68.9 (10.8)	71.7 (11.4)	<0.0001	66.4 (10.4)	68.7 (11.4)	71.2 (12.2)	0.0007
BMI (kg/m^2^)	24.4 (3.1)	25.1 (7.9)	25.7 (3.1)	<0.0001	24.6 (3.0)	25.1 (3.4)	25.6 (3.3)	0.0119
Duration of DM (years)	4.6 (4.5)	4.1 (4.2)	4.1 (3.9)	0.0369	4.0 (4.3)	3.8 (4.1)	4.4 (4.4)	0.4291
HbA_1c_ (%)	7.2 (0.9)	7.2 (0.9)	7.3 (0.9)	0.0358	7.2 (0.8)	7.2 (0.8)	7.3 (0.8)	0.5528
FPG (mmol/L)	7.4 (1.9)	7.5 (1.7)	8.0 (2.0)	<0.0001	7.3 (1.7)	7.5 (1.6)	7.8 (1.9)	0.0225
120 min PPG (mmol/L)	11.8 (3.6)	12.6 (3.3)	13.3 (3.8)	0.013	11.0 (3.5)	12.8 (3.5)	11.6 (3.8)	0.1754
TG (mmol/L)	0.95 (0.23)	1.63 (0.21)	3.44 (2.11)	<0.0001	0.97 (0.21)	1.62 (0.22)	3.36 (2.12)	<0.0001
Total cholesterol (mmol/L)	4.37 (0.87)	4.78 (0.83)	5.04 (0.93)	<0.0001	4.42 (0.90)	4.88 (0.89)	5.03 (0.98)	<0.0001
SBP (mmHg)	125 (11)	126 (11)	126 (11)	0.1743	125 (12)	126 (12)	127 (12)	0.4498
DBP (mmHg)	77 (8)	77 (7)	78 (8)	0.0030	77 (7)	77 (8)	79 (7)	0.0366
ALT (U/L)	22 (13)	26 (14)	29 (15)	<0.0001	21 (10)	25 (13)	28 (14)	<0.0001
Plasma creatinine (*μ*mol/L)	64 (15)	65 (16)	66 (15)	0.0286	64 (16)	64 (15)	66 (17)	0.4972

All data except for the gender were in mean (standard deviation (SD)). FAS: full analysis set; VLDM: vildagliptin (50 mg bid) plus metformin (500 mg bid); HDM: metformin uptitration (1000 mg bid); 1^st^ tertile: TG < 1.28 mmol/L; 2^nd^ tertile: 1.28 mmol/L ≤ TG ≤ 2.03 mmol/L; 3^rd^ tertile: TG > 2.03 mmol/L; TG: triglyceride; BMI: body mass index; FPG: fasting plasma glucose; 120 min PPG: 120-minute postprandial blood glucose; SBP: systolic blood pressure; DBP: diastolic blood pressure; ALT: alanine aminotransferase.

**Table 2:** *Δ*HbA_1c_ at weeks 12 and 24 for patients of the 3 TG-based tertiles in the VLDM and HDM groups (FAS LOCF).

*Δ*HbA_1c_	VLDM				HDM			
	1^st^ tertile	2^nd^ tertile	3^rd^ tertile	*P*	1^st^ tertile	2^nd^ tertile	3^rd^ tertile	*P*
Week 12								
Sample size (*n*)	799	837	798	—	168	154	150	—
Mean ± SD (%)	-0.55 ± 0.82	-0.52 ± 0.75	-0.48 ± 0.79	—	-0.33 ± 1.03	-0.25 ± 0.88	-0.22 ± 0.79	—
*Univariate analysis*								
LSMean (SE) (%)	-0.56 ± 0.03	-0.53 ± 0.02	-0.45 ± 0.03^∗^^*ς*^	0.007	-0.35 ± 0.07	-0.25 ± 0.07	-0.20 ± 0.07	0.332
*Multivariate analysis*								
LSMean (SE) (%)	-0.57 ± 0.03	-0.52 ± 0.02	-0.45 ± 0.03^∗^^*ς*^	0.008	-0.34 ± 0.07	-0.25 ± 0.07	-9	0.503
Week 24								
Sample size (*n*)	808	847	804	—	169	154	148	—
Mean ± SD (%)	-0.57 ± 0.91	-0.55 ± 0.86	-0.51 ± 0.88	—	-0.49 ± 0.85	-0.39 ± 0.82	-0.31 ± 0.82	—
*Univariate analysis*								
LSMean (SE) (%)	-0.58 ± 0.03	-0.56 ± 0.03	-0.48 ± 0.03^∗^^*ς*^	0.025	-0.51 ± 0.06	-0.38 ± 0.06	-0.30 ± 0.07	0.064
*Multivariate analysis*								
LSMean (SE) (%)	-0.57 ± 0.03	-0.56 ± 0.03	-0.49 ± 0.03	0.113	-0.49 ± 0.06	-0.38 ± 0.06	-0.32 ± 0.07	0.184

^∗^3^rd^ tertile vs. 1^st^ tertile: *P* < 0.05; ^*ς*^3^rd^ tertile vs. 2^nd^ tertile: *P* < 0.05. 1^st^ tertile: TG < 1.28 mmol/L; 2^nd^ tertile: 1.28 mmol/L ≤ TG ≤ 2.03 mmol/L; 3^rd^ tertile: TG > 2.03 mmol/L; *Δ*HbA_1c_: glycated hemoglobin (HbA_1c_) change from baseline; VLDM: vildagliptin (50 mg bid) plus metformin (500 mg bid); HDM: metformin uptitration (1000 mg bid); FAS: full analysis set; LOCF: last observation carried forward; LSMean: least square mean. The univariate analysis used the TG subgroups as the variable and baseline HbA_1c_ as the covariate.

**Table 3:** Relationship between baseline characteristics and *Δ*HbA_1c_ in the VLDM and HDM groups by multivariate linear regression analysis (FAS LOCF).

	VLDM		HDM	
	*B* (95% CI)	*P* value	*B* (95% CI)	*P* value
Week 12				
Log (baseline TG)	0.1083 (0.0537, 0.1629)	<0.001	0.0800 (-0.0821, 0.2422)	0.333
Plasma creatinine	-0.0035 (-0.0053, -0.0017)	<0.001	-0.0052 (-0.0104, -0.0000)	0.052
Duration of DM	0.0231 (0.0163, 0.0298)	<0.001	0.0083 (-0.0112, 0.0279)	0.403
Baseline HbA_1c_	-0.4127 (-0.4455, -0.3799)	<0.001	-0.2582 (-0.3620, -0.1544)	<0.001
Week 24				
Log (baseline TG)	0.0551 (-0.0071, 0.1173)	0.082	0.0761 (-0.0691, 0.2212)	0.304
Plasma creatinine	-0.0036 (-0.0057, -0.0016)	<0.001	-0.0014 (-0.0061, 0.0032)	0.541
Duration of DM	0.0234 (0.0157, 0.0311)	<0.001	0.0314 (0.0139, 0.0490)	<0.001
Baseline HbA_1c_	-0.4290 (-0.4663, -0.3917)	<0.001	-0.3325 (-0.4248, -0.2402)	<0.001

TG: triglyceride; FAS: full analysis set; LOCF: last observation carried forward; VLDM: vildagliptin (50 mg bid) plus metformin (500 mg bid); HDM: metformin uptitration (1000 mg bid); 95% CI: 95% confidence interval; HbA_1c_: glycated hemoglobin; TC: total cholesterol.

## Data Availability

The previously reported VISION data were used to support this study and are available at DOI 10.1111/dom.12667. These datasets are available from the corresponding author upon request.
